# Syngas Production via Oxidative Reforming of Propane Using a CO_2_- and O_2_-Permeating Membrane

**DOI:** 10.3390/membranes14110238

**Published:** 2024-11-12

**Authors:** José A. Fabián-Anguiano, Lorena G. Cuéllar-Herrera, José A. Romero-Serrano, Issis C. Romero-Ibarra, Antonieta García-Murillo, Felipe Carrillo-Romo, José Ortiz-Landeros

**Affiliations:** 1 Instituto Politécnico Nacional, Escuela Superior de Ingeniería Química e Industrias Extractivas, Departamento de Ingeniería en Metalurgia y Materiales, UPALM-Zacatenco, IPN Avenue, Mexico City 07738, Mexico; jfabiana1000@alumno.ipn.mx (J.A.F.-A.); lcuellarh1000@alumno.ipn.mx (L.G.C.-H.); aromeros@ipn.mx (J.A.R.-S.); 2 Instituto Politécnico Nacional, Unidad Profesional Interdisciplinaria en Ingeniería y Tecnologías Avanzadas, IPN Avenue, Mexico City 07340, Mexico; iromero@ipn.mx; 3 Instituto Politécnico Nacional, Centro de Investigación e Innovación Tecnológica, Cerrada de Cecati S/N, Santa Catarina, Azcapotzalco, Mexico City 02250, Mexico; angarciam@ipn.mx (A.G.-M.); fcarrillo@ipn.mx (F.C.-R.)

**Keywords:** membrane, CO_2_ separation, O_2_ separation, oxidative reforming of propane, syngas

## Abstract

Recently, ceramic–carbonate membrane reactors have been proposed to selectively separate CO_2_ at elevated temperatures and to valorize this pollutant gas by coupling a catalyzed reaction. This work explores using a membrane reactor to perform the oxidative reforming of propane by taking advantage of the CO_2_- and O_2_-permeating properties of a LiAlO_2_/Ag–carbonate membrane. The fabricated membrane showed excellent permeation properties, such as CO_2_/N_2_ and O_2_/N_2_ selectivity, when operating in the 725–850 °C temperature range. The membrane exhibited remarkable stability during the long-term permeation test under operating conditions, exhibiting minor microstructural and permeation changes. Then, by packing a Ni/CeO_2_ catalyst, the membrane reactor arrangement showed efficient syngas production, especially at temperatures above 800 °C. A hydrogen-rich syngas mixture was obtained by the contributions of the oxidative reforming and cracking reactions. Specific issues observed regarding the membrane reactor’s performance are attributed to the catalyst that was used, which experienced significant poisoning by carbon deposition during the reaction, affecting syngas production during the long-term test. Thermodynamic calculations were performed to support the experimental results.

## 1. Introduction

Over the last 20 years, liquid fuel production, as a primary energy source, has increased significantly to cover global industrial demands [1]. Fischer–Tropsch synthesis is a well-known process to synthesize fuels and other chemical products; this technology can produce ammonia, dimethyl ether, and low-carbon olefins such as ethylene and propylene from non-petroleum resources; for instance, syngas, a mixture of H_2_ and CO [2,3,4]. The key point is that syngas can be derived by converting light hydrocarbons with CO_2_, reducing greenhouse gas emissions from conventional industrial processes. Recently, reforming processes for hydrocarbons with CO_2_ have been reinvestigated because of the potential to obtain syngas mixtures exhibiting different stoichiometric ratios of H_2_/CO. For example, dry reforming of CH_4_ with CO_2_ has been used a case study for obtaining a low syngas ratio of about ~1 [5]. Like CH_4_ dry reforming, propane (C_3_H_8_) reforming has recently attracted attention as a new route for decarbonization. Propane occurs during the petroleum refining process, and it is produced in a liquid state at room temperature under pressure; moreover, its storage and distribution are practical. Based on the above, hydrogen production through propane reforming has been raised [6,7,8]; specifically, the reaction of C_3_H_8_ with CO_2_ is currently being investigated. Propane reforming can guarantee a syngas ratio of about 1.5, as shown in the following Reaction (1) [9].

(1) $$C_{3}H_{8}+{3CO}_{2}\to 6CO+{4H}_{2}∆H^{{}^{\circ}}=621.3\;{kJ\cdot mol}^{-1}$$

The main drawback of a dry reforming reaction is that it is highly endothermic and, therefore, energy-consuming. The utilization of air/oxygen mixtures has been investigated through the so-called oxidative reforming reaction by combining a partial oxidation process, as represented by Reaction (2), to decrease the energy costs and produce syngas during C_3_H_8_ reforming with CO_2_ (Reaction (3)) [8,10,11].

(2) $$C_{3}H_{8}+{1.5O}_{2}\to 3CO+{4H}_{2}∆H^{{}^{\circ}}=-227.7\;{kJ\cdot mol}^{-1}$$

(3) $$C_{3}H_{8}+1.5{CO}_{2}+0.75O_{2}\to 4.5CO+4H_{2}∆H^{{}^{\circ}}=196.7\;{kJ\cdot mol}^{-1}$$

In the state of the art, the reforming of catalysts based on precious metal and metal oxides has gained attention in propane reforming reactions because these materials can show a selective transformation [12]. However, the catalysts’ lifetimes, costs, and performances are currently still subjects of study [13]. In recent studies, catalytic membrane reactors have been proposed to produce syngas through reforming and other related reactions; these technologies rely on the design and fabrication of an appropriate membrane that offers good separation performance, high thermal and chemical stabilities, and high selectivity toward obtaining the gaseous species of interest, for example, using CO_2_ over N_2_ for its subsequent conversion into syngas [14,15]. Therefore, catalytic membrane reactors have emerged as a new technology to carry out the gas separation process and chemical reactions simultaneously. Different approaches to developing catalytic membrane reactors have been considered in studying a new class of membranes based on supported molten salts, e.g., metal–carbonates and ceramic–carbonate membranes [16,17,18]. For example, silver–molten-carbonate membranes have been investigated to selectively separate CO_2_ at high temperatures. Silver exhibits the highest electrical conductivity among metals and negligible chemical reactivity against alkaline molten- salt compositions [19]. Using these properties, supported molten-salt membranes based on silver can simultaneously separate CO_2_ and O_2_ with high permeation fluxes of 0.82 and 0.43 mL·min^−1^·cm^−2^ at intermediate temperatures (650–700 °C), respectively [19]. Briefly, the species transport in this kind of membrane is based on the surface formation and subsequent ionic conduction of CO_3_^2−^ through the bulk of the carbonate phase. The proposed mechanism has been extensively described previously [16]. Briefly, the mechanism involves the electronic conductivity of the solid phase in the membrane according to Reaction (4).

(4) CO2 g+12O2 +2 e−↔CO32−

Recently, various attempts to improve the thermal stability of silver–molten-carbonate membranes have included the surface modification of the silver matrix, for example, with ceramics using conventional impregnation methods and sophisticated techniques, such as chemical dealloying, chemical vapor deposition, and atomic layer deposition [20,21,22,23]. The refractory properties of the deposited metal oxides and the effectiveness of the surface protection at the silver matrix’s nanometric scale have improved the membrane’s thermal stability and stabilized the permeation flux at high temperatures. More recently, the fabrication of cermets by integrating silver with ceramic oxides, such as SDC and LiAlO_2_, has been subject to research on developing supported molten-salt membranes [24,25]. Using SDC and γ-LiAlO_2_ as ceramic fillers improves the wettability properties of the silver matrix against the molten-carbonate phase and hinders its thermal densification at high temperatures. These features guarantee the highly selective separation of CO_2_ and O_2_ at high temperatures, reaching CO_2_-permeation flux values of as high as 0.49 and 0.78 mL·min^−1^·cm^−2^ for SDC/Ag and γ-LiAlO_2_/Ag molten-salt membranes at 850 °C, respectively. Moreover, cermet–molten-salt membranes extend the operational lifetime, taking the conventional silver–molten-carbonate membranes as a baseline [19,20,21,22,23]. The improvement in the wettability properties against molten carbonates and the protraction of the operating time of the silver-based membranes has opened the possibility to explore their use in hydrogen production by coupling reforming reactions [25,26]. For example, cermet–molten-salt membranes, composed of γ-LiAlO_2_/Ag and a molten-carbonate mixture of Li_2_CO_3_, Na_2_CO_3_, and K_2_CO_3_, have been studied to obtain syngas through the oxidative methane reforming reaction. The membrane is shown to be highly stable under reaction conditions for up to 120 h, achieving a total syngas production (H_2_+CO) of 4.94 mL·min^−1^·cm^−2^ [25]. Taking advantage of the high performance of cermet–molten-carbonate membranes, in the present work, a γ-LiAlO_2_/Ag-based membrane was exploited to elucidate the feasibility of obtaining syngas through the oxidative reforming of propane at elevated temperatures (725–850 °C).

## 2. Materials and Methods

### 2.1. Membrane Preparation and Characterization

To obtain the cermet–molten-carbonate membrane, first, cermet powders of γ-LiAlO_2_/Ag were prepared from Ag atomized commercial powders of 400 mesh (99.9%, Stannum, Mexico City, Mexico) and γ-LiAlO_2_ ceramic powders; the latter were chemically synthesized using the EDTA/citrate method, as previously described in detail [25,27]. Cermet powders with a composition of 50:50 vol. % were prepared by ball milling using a Spex-Mixer/Mill apparatus (Chemplex Instruments, Urbana, IL, USA). The grinding medium was alumina balls, using a ratio of balls to powders of 10 to 1 in weight. The powders were dry milled for 15 min, recovered from the milling jar, and mixed with polyvinyl alcohol as a polymeric binder to subsequently be uniaxially pressed into disk-shaped membrane supports. An incipient sintering process was conducted at 900 °C for 20 h using a heating and cooling rate of 3 °C/min to obtain porous supports with a final porosity of around 33 vol. %. Finally, a dense membrane was obtained by direct infiltration of the supports with a eutectic ternary mixture of Li_2_CO_3_/Na_2_CO_3_/K_2_CO_3_ using a molar ratio of 43.5/31.5/25, respectively, at 550 °C. After infiltration, the cermet–molten-carbonate membrane was slowly cooled, and the carbonate excess was removed using sandpaper until a final membrane thickness of 1.2 mm.

The membrane was characterized by the X-ray diffraction (XRD) technique to identify the crystalline phase structure; for this purpose, the diffractometer model D8 Focus (Bruker, Billerica, MA, USA), equipped with a Cu-Ka radiation source, was used. The XRD diffraction patterns were recorded in a 2-theta range of 10–90° using a scanning rate of 2° min^−1^ and a step size of 0.02°. The morphology and microstructural features of the samples were characterized using a JSM-6400 scanning electron microscope (SEM) from JEOL, Tokyo, Japan. The analyses were conducted using both secondary electron and backscattered-electron techniques. Archimedes measurements, based on the international standard ASTM C373-88 [28], were performed to determine the effective pore volume of the supports. In addition, in the unsteady state, He-permeation measurements were also used to test the open and interconnected porosity in the supports and to corroborate the membrane’s infiltration by molten carbonates.

### 2.2. Permeation Measurements and Oxidative Reforming of Propane Test

The CO_2_- and O_2_-permeation measurements were conducted using a commercial Probostat high-temperature permeation system (NORECs, Norway) equipped with gas mixers and flow controllers, as described in previous works [14,25]. Briefly, the membrane was tightly attached to an inner alumina tube with a silver seal (99% purity) using special spring accessories. The system was completely enclosed with an outer highly dense alumina tube and heated at 950 °C using a ramping rate of 2° min^−1^ to soften the silver seal and, therefore, the membrane sealing. The system was fed with a gas mixture composition of CO_2_/O_2_/N_2_ in a 15/17/68 vol. % and a constant flow rate of 50 mL·min^−1^. N_2_ was used as a diluent to monitor the presence of leaks through the membrane defects. The permeation measurements were conducted in a temperature range from 725 to 850 °C with increases of 25 °C. Each reading was recorded when the system reached steady state conditions after 60 min of reaching the target temperature. On the permeate side, a constant argon flow rate (50 mL·min^−1^) was used as a sweep gas to sweep the permeated species.

To perform the oxidative reforming reaction in the system, a catalyst bed of 10 wt.% Ni supported on CeO_2_ was placed 1 cm underneath the membrane on the sweep side. In the membrane’s permeate side, the sweep gas was switched from pure Ar to a mixture of 5 vol. % of C_3_H_8_ in Ar with a total flow of 50 mL·min^−1^. This propane concentration means an excess of the stoichiometric amount of this gas required to perform the oxidative reforming (Reactions (3) and (4)) based on the CO_2_- and O_2_-permeation measurements. Before the reaction test, the catalyst was held under a gas mixture of 50/50 vol. % H_2_ and Ar at 850 °C for 60 min to reduce the Ni active phase. The permeate species and the reaction products were continuously analyzed in the whole range of temperatures studied by gas chromatography, using a chromatograph model GC-2014 (SHIMADZU, Kyoto, Japan) equipped with a TCD detector and a Carboxen-1010 PLOT capillary column. Moreover, in situ analysis by an online FT-IR was coupled with the sweep gas effluent to monitor the presence of water concentration during the reaction test. For this purpose, an FT-IR equipment model ALPHA II (Bruker, USA) was used.

The permeation flux of CO_2_ and O_2_ were calculated considering the total flux and the N_2_ leak contribution by the Knudsen diffusion (if the case); however, if N_2_ was not detected, a minimum lake value of 0.0013 mL·min^−1^·cm^−2^ was considered for the correction of the fluxes for CO_2_ and O_2_ based on the chromatograph detection limit. In the same sense, the conversion rates for C_3_H_8_, CO_2_, and O_2_ were estimated based on the chromatography analysis (Supplementary Materials). Thermogravimetric analysis of the fresh and spent catalysts was performed in a dry air atmosphere using a thermobalance model Regulus STA 2500 (Netzsch, Waldkraiburg, Germany). Finally, to elucidate the syngas production through the oxidative reforming of propane, thermodynamic calculations were conducted using the FactSage 7.2 thermochemical software [29].

## 3. Results and Discussion

### 3.1. Synthesis and Characterization of γ-LiAlO_2_/Ag Powders and Fabrication of Cermet–Molten-Carbonate Membranes

**Figure 1:**
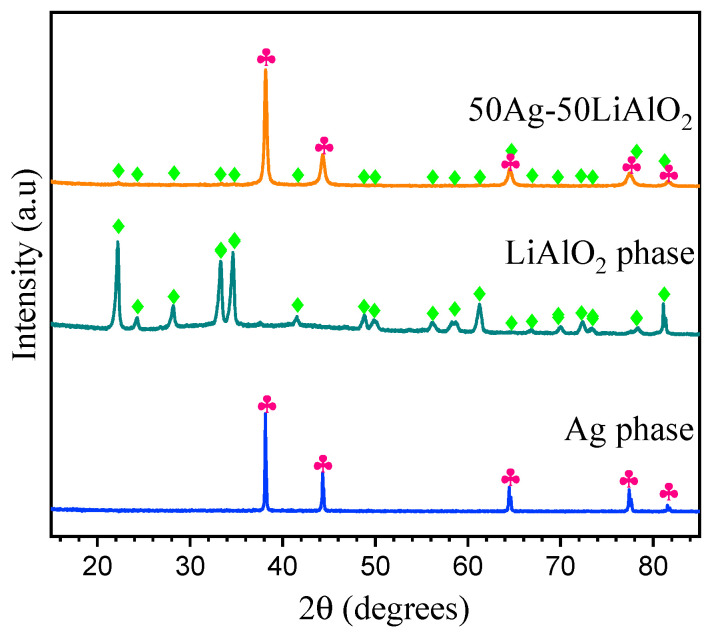
XRD analysis of the pristine materials (γ-LiAlO_2_, Ag) and γ-LiAlO_2_/Ag cermet (50:50 vol. %) synthetized by the ball milling process.

Figure 1 shows the XRD analysis of the γ-LiAlO_2_/Ag cermet prepared by the ball milling process. The γ-LiAlO_2_/Ag diffraction peaks indicate that both cermet components are highly stable during milling because neither structural changes nor secondary phases were identified because of cermet formation starting from the single components. It is notable that the reflection peaks of the ceramic phase are less intense in relation to the metallic phase; this fact can be attributed to the difference in the atomic weights of the elements present in each phase of the cermet and, therefore, the X-ray mass absorption coefficient of the pristine materials [25,30]. The characteristic reflection peaks of the materials were identified using the ICSD database. For the γ-LiAlO_2_ phase, a PDF 98-003-0249 card was used, and it matched a *P41212* space group, whereas, for silver, the PDF 98-006-4994 card with an *Fm3m* space group was used.

**Figure 2:**
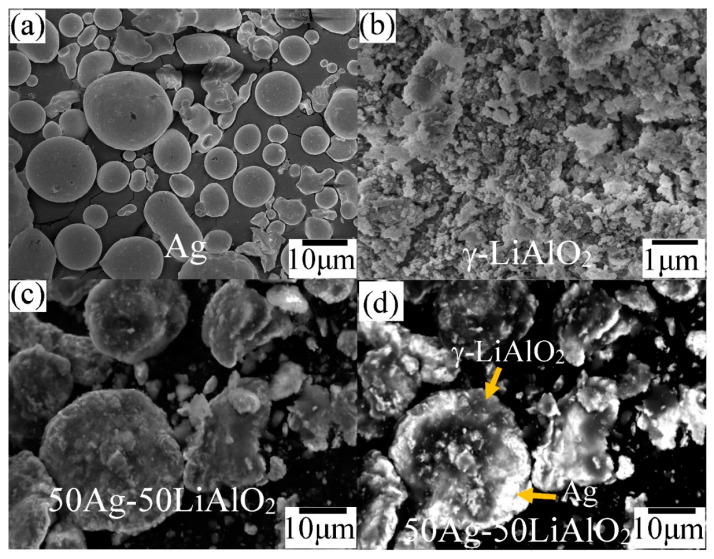
Microstructural analyses by SEM of (**a**) silver, (**b**) γ-LiAlO_2_, and (**c**,**d**) γ-LiAlO_2_/Ag cermet powders (50:50 vol. %) synthetized by the milling process.

The morphology, particle size, and microstructural features of the γ-LiAlO_2_/Ag cermet differ significantly from the pristine materials (Figure 2a–c). On the one hand, the silver powders were mainly composed of spheroidal particles exhibiting particle sizes in a range of 4–35 μm; on the other hand, γ-LiAlO_2_ was composed of soft, irregular agglomerates on the submicrometric scale. In the case of cermet, the powders showed irregular morphologies and particle sizes of about 5–30 μm. Finally, the SEM image obtained by the backscattering technique (Figure 2d) shows that milling processing promotes the integration of both single materials, ceramic, and metal to form the expected composite. This technique agrees with the results of the XRD, since it successfully indicated the formation of the cermet. Similar behavior was observed during the formation of the Ce_0.9_Sm_0.1_O_2−δ/_Ag and LiAlO_2_/Ag cermets, which have previously been studied [24,25]; therefore, these results evidence the feasibility and reproducibility of the ball milling procedure for cermets’ formation.

**Figure 3:**
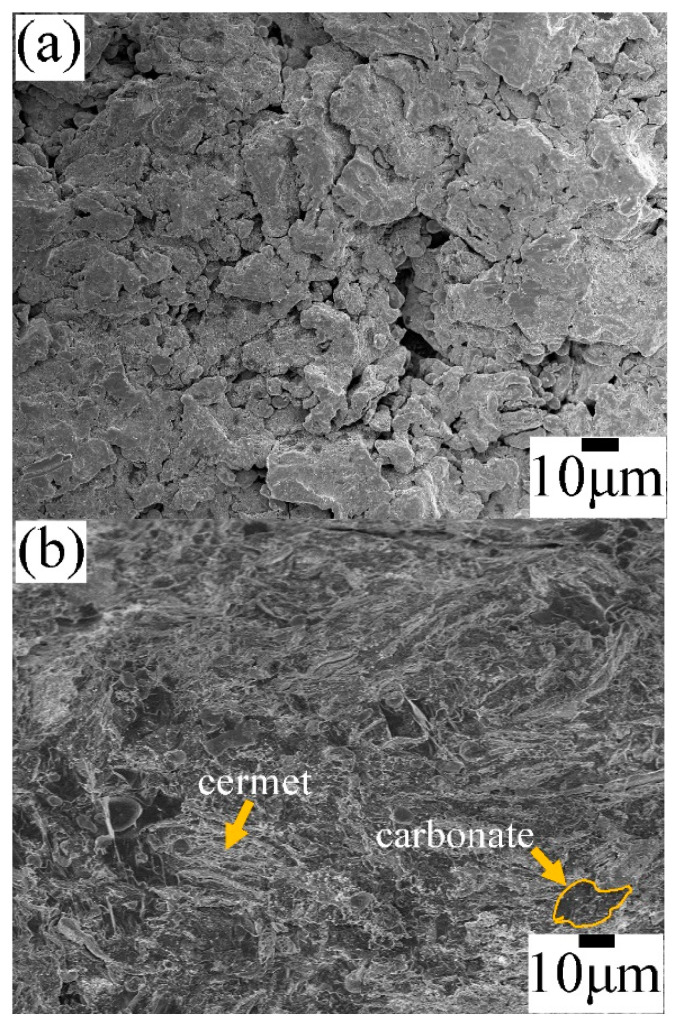

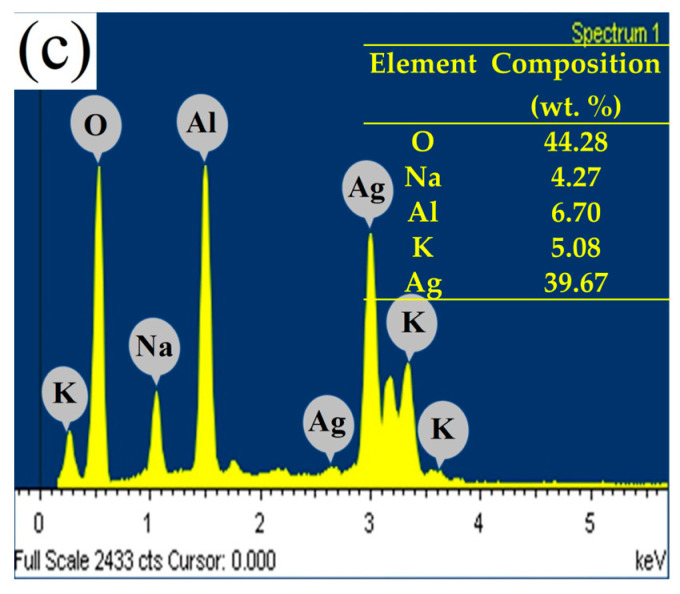
Microstructural analyses by SEM: (**a**) porous support sintered at 900 °C for 20 h; (**b**) cermet–molten-carbonate membrane obtained by infiltration of the Li_2_CO_3_/Na_2_CO_3_/K_2_CO_3_ mixture; (**c**) EDS spectrum shown as evidence of the carbonates’ incorporation into the membrane.

Figure 3 shows a SEM image of the cross-section of the porous support obtained by pressing and subsequent sintering at 900 °C, as well as the cermet–molten-carbonate membrane obtained after infiltration. As shown in Figure 3a, the cermet support has a highly porous microstructure, consisting of different irregular pore sizes. This porosity represents an open porosity of around 33 vol. %, estimated by Archimedes’ method. Moreover, the steady-state helium permeation test indicated that the porosity-to-tortuosity ratio $\left(\frac{\varepsilon }{\tau }\right)$ of the cermet support was 0.27, with a helium permeance value of 2.24 × 10^−5^ mol·Pa^−1^·m^−2^·s^−1^. These results suggest that the porosity of the support is also highly interconnected. Figure 3b shows details of the cross-section of the infiltrated membrane. In this case, the open porosity of the support was wholly filled with the molten-carbonate phase, leading to a dense microstructure typical for this type of membrane. In addition, the helium-permeation measurement corroborated a successful densification process and indicates that the membrane is free of defects, because the permeance value decreased to 0.94 × 10^−10^ mol·Pa^−1^·m^−2^·s^−1^, which means five orders of magnitude lower than the porous support. Figure 3c shows the EDS spectrum of the infiltrated sample. The observed signals evidence the presence of all of the chemical constituents after infiltration.

### 3.2. Permeation Measurements and Oxy-CO_2_ Reforming of Propane at High Temperatures in the Membrane Reactor Arrangement

**Figure 4:**
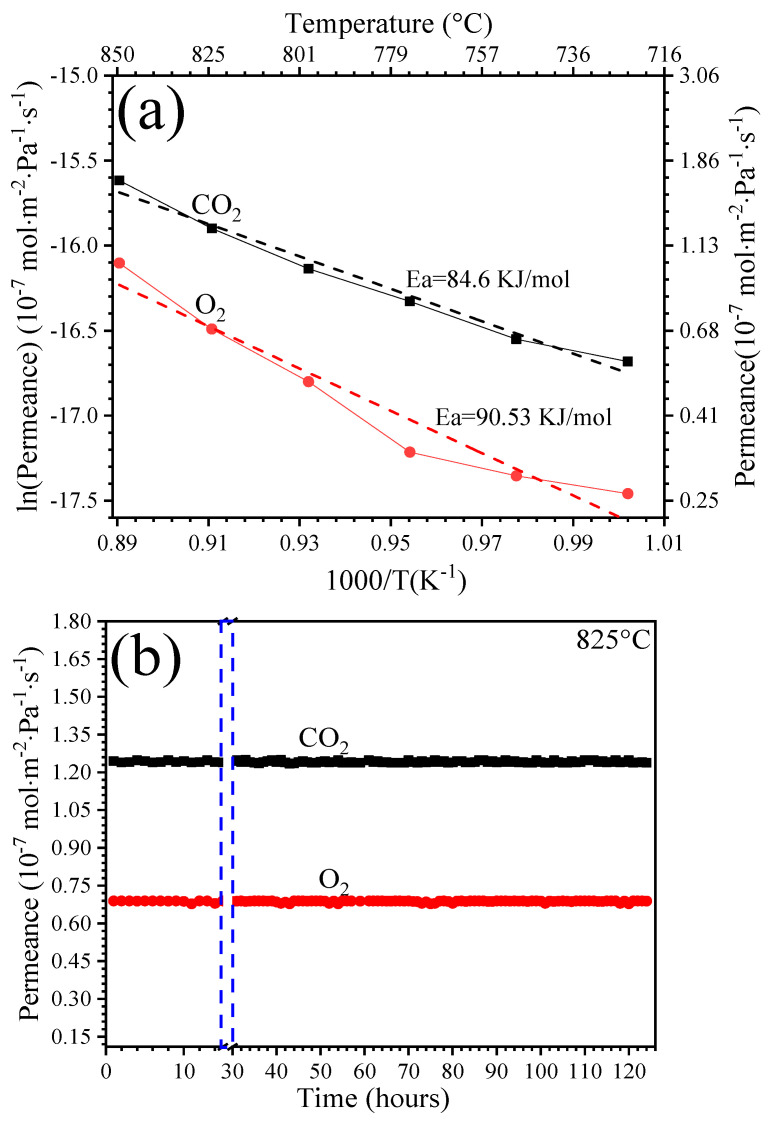
CO_2_- and O_2_-permeation measurements at high temperature of the cermet–molten-carbonate membrane: (**a**) CO_2_- and O_2_-permeance properties as a function of temperature after the first 24 h of stabilization; (**b**) long-term stability of the membrane under permeance conditions at 825 °C. Dash lines indicate the period wherein the measurements against temperature where performed.

Figure 4 shows the permeance properties of the dense membrane in a temperature range of 725–850 °C. Before carrying out a series of measurements as a function of temperature, a stabilization period was maintained in the membrane for the first 24 h at 825 °C (Figure 4b). Afterward, the permeance data were recorded once it was corroborated that the CO_2_ and O_2_ fluxes were stable. As shown in Figure 4a, the CO_2_ and O_2_ permeations through the membrane increased because of the ionic conduction of the surface-formed CO_3_^2−^ species (Reaction (4)), which relies on the ionic conduction properties of the molten-carbonate phase, that is, a thermally activated process [14]. Overall, the CO_2_ and O_2_ transport was remarkable—between 725 and 825 °C—and the permeance increased from 0.56 × 10^−7^ to 1.65 × 10^−7^ mol·Pa^−1^·m^−2^·s^−1^ for CO_2_ and from 0.26 × 10^−7^ to 1.01 × 10^−7^ mol·Pa^−1^·m^−2^·s^−1^ for O_2_, respectively. In the whole temperature range studied, no N_2_ concentration was detected, i.e., a negligible leak was observed. Hence, considering the detection limit of the GC, if a minimum concentration of N_2_ is present, this would be equivalent to a permeation value of 0.4 × 10^−10^ mol·Pa^−1^·m^−2^·s^−1^, which would not change significantly the observed membrane selectivity.

As expected, the apparent activation energy values were calculated by constructing Arrhenius plots (Figure 4a). The CO_2_ and O_2_ permeances had similar *E_a_* values of 84.6 kJ·mol^−1^ for CO_2_ and 90.5 kJ·mol^−1^ for O_2_. This fact results from the transport of both species being a concurrent phenomenon related to Reaction (4) (backward). The long-term stability of the membrane under permeation conditions was examined at 825 °C.

Figure 4b shows the permeation experiments as a function of time. When the temperature reached 825 °C and was maintained for the next 95 h, the CO_2_- and O_2_-permeance fluxes were highly stable, at this point completing a total of 125 h under operating conditions. Remarkably, the membrane achieved concomitant separation with a total permeance value of $J_{{CO}_{2}}+J_{O_{2}}$ equal to 2.66 × 10^−7^ mol·Pa^−1^·m^−2^·s^−1^ at 825 °C.

**Figure 5:**
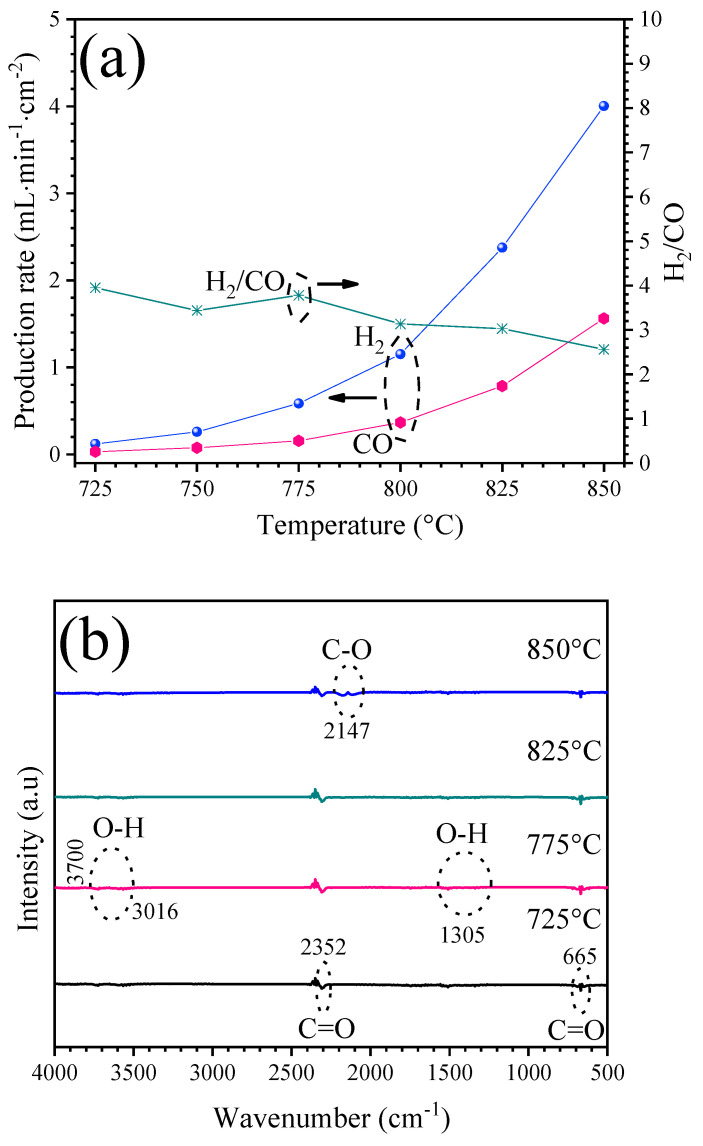
Syngas production when using a sweep of 5 vol. % of C_3_H_8_ in argon: (**a**) H_2_ and CO production rates as a function of temperature; (**b**) FT-IR in situ analysis of the syngas at different temperatures. Arrows indicate the corresponding Y axis.

As mentioned, at least on the laboratory scale, the cermet–molten-carbonate membrane showed high thermal and chemical stability over the long term. Owing to this feature, the oxidative reforming of propane was conducted using the same membrane. Figure 5 shows the syngas production obtained when C_3_H_8_ diluted in Ar was fed as the sweep gas in the experimental arrangement to perform the reaction. As shown in Figure 5a, the H_2_ production rate improved as the temperature increased, achieving a flux rate from 0.12 to 4.0 mL·min^−1^·cm^−2^ when the membrane reached 850 °C. Similarly, the CO increased as a function of the temperature, reaching a total production rate of 1.56 mL·min^−1^·cm^−2^ at 850 °C. Considering that the excess of propane present in the sweep was consumed, it can be concluded that the amount of hydrogen produced was higher than predicted by the stoichiometric oxidative reforming reactions described in Equations (3) and (4), and, therefore, other side reactions must be involved. As a result, the H_2_/CO ratio was about 3.9 at lower temperatures (725 °C), whereas it was 2.5 at 850 °C. Actually, based on the GC analysis, the presence of C_3_H_8_, CO_2_, and O_2_ was not detected in the sweep out for the whole temperature range studied, which means the total conversion of the reactants was achieved.

A complementary FT-IR analysis in situ was also used to analyze the sweep gas composition. In Figure 5b, the FT-IR spectrum at different temperatures shows very weak bands of trace species. The O-H from water molecules was between 3016 and 3700 cm^−1^ and symmetric and asymmetric stretching of CO_2_ occurred at 665 and 2352 cm^−1^. On the other hand, the FT-IR spectrum shows bands at 2147 cm^−1^ assigned to the stretching vibrational modes of the CO. Therefore, the results suggest that propane was consumed to favor syngas production through both dry reforming and partial oxidation reactions at high temperatures, but a reverse water–gas shift reaction may be involved in syngas production, as has also been reported for oxygen-transporting membranes studied under oxidative reforming of propane [31,32].

**Figure 6:**
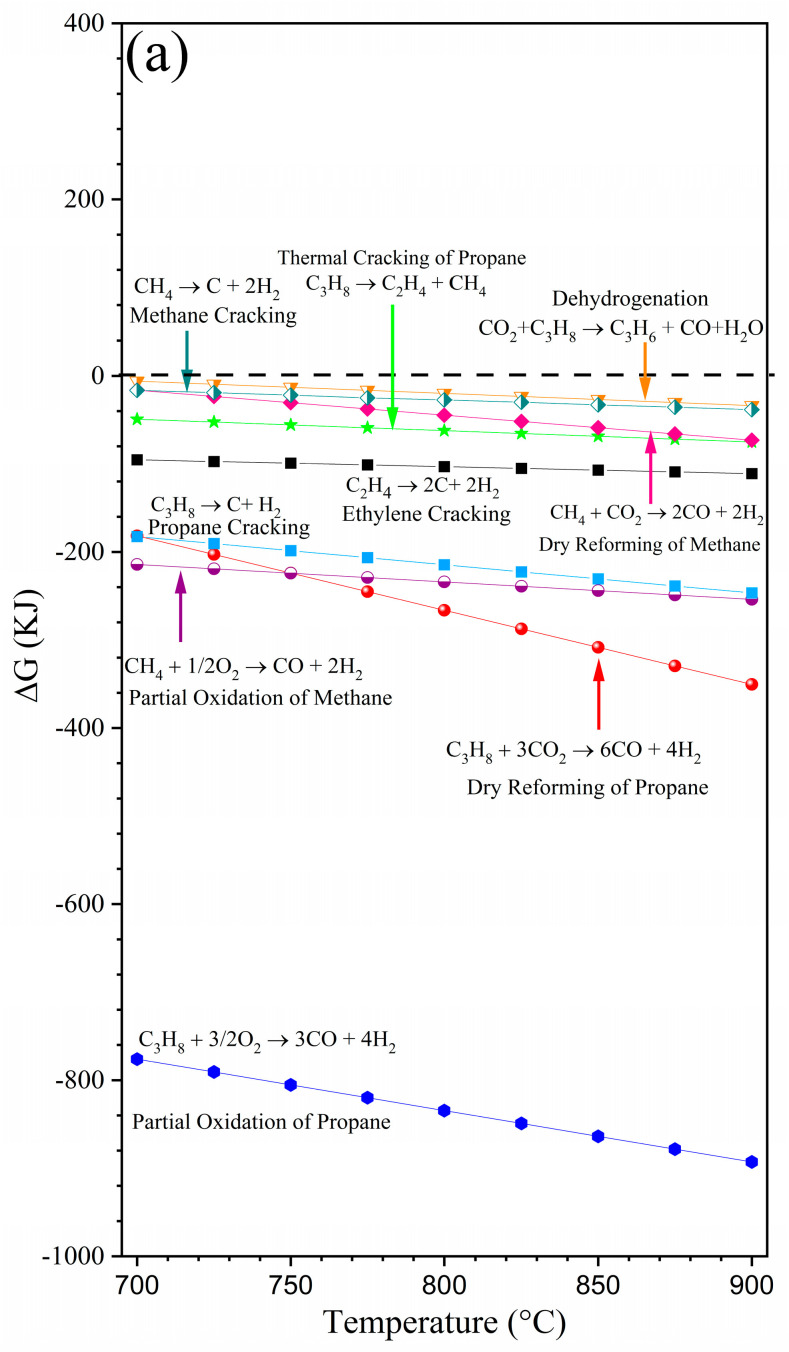

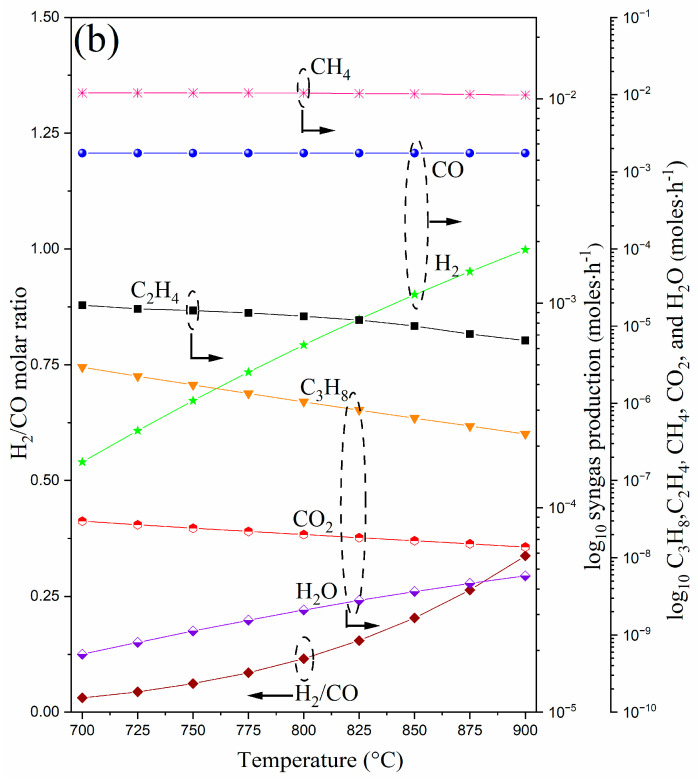
Thermochemical simulations of syngas using FactSage 7.2 software: (**a**) ΔG° versus temperature of the main reactions involved in the syngas production; (**b**) thermodynamic equilibrium calculus of the produced species at high temperatures (600–900 °C). Arrows indicate the corresponding Y axis.

Afterward, thermochemical simulations of syngas were conducted using the FactSage 7.2 software [29]. As shown in Figure 6a, oxidative reforming of propane is highly viable, following both the partial oxidation and dry reforming reactions in the temperature range at which the membrane was studied. Furthermore, thermodynamic calculations support the possible influence of other reactions occurring during syngas production. Different reaction pathways can also involve thermal dehydrogenation, thermal cracking, complete combustion of propane, and even methanation and partial oxidation of methane, as reported in [11,31,32,33,34].

On the other hand, the equilibrium composition of the syngas was calculated as a function of the temperature (Figure 6b). The thermodynamic equilibrium calculus indicates that, certainly, when the temperature increases from 700 to 900 °C, CO_2_ and C_3_H_8_ are consumed to favor syngas production. However, based on thermodynamics, CO production was higher than hydrogen for the whole temperature range. This fact does not match the experimental data, and it could be attributed to different factors, including catalyst performance in terms of catalyst selectivity and significant contributions by the cracking reactions that can result in an increase in the H_2_ yield and, therefore, the H_2_/CO ratio observed experimentally. Moreover, thermodynamic estimations also suggest the formation of small quantities of water, corroborating the experimental data from this work.

### 3.3. Stability Test Under Oxidative Reforming of Propane

**Figure 7:**
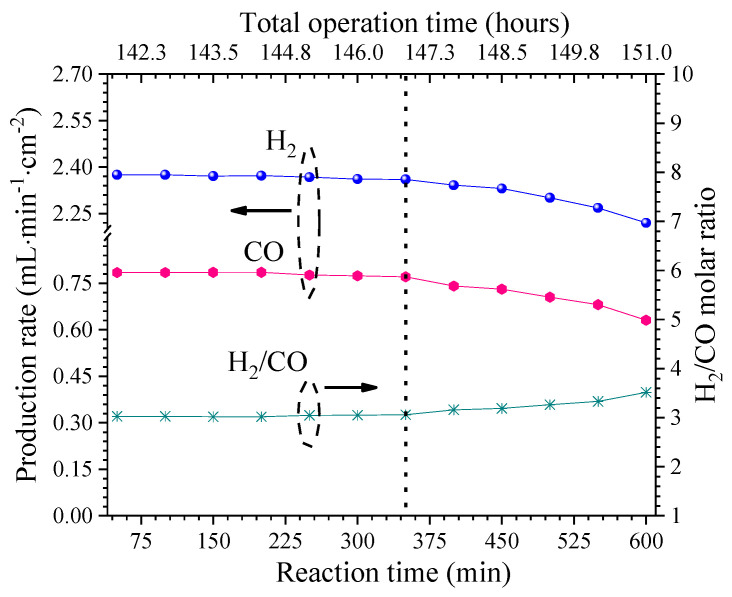
Stability of syngas production through the cermet–molten-carbonate membrane under oxidative reforming of propane. Stability of membrane with a total continuous operation time of 151 h. Arrows indicate the corresponding Y axis.

**Figure 8:**
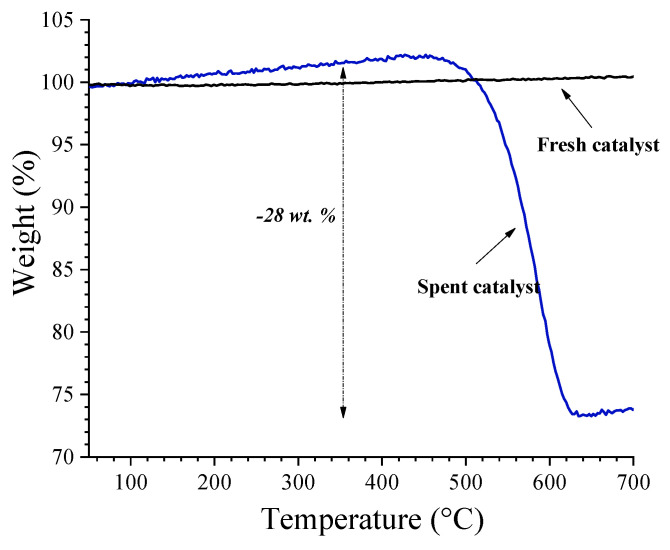
TGA analysis of the fresh and spent catalysts. The weight change indicates the removal by combustion of the carbon formed due to the thermal cracking reactions.

The membrane was kept under reaction conditions for 10 h longer at 825 °C, for a total of 151 h of operation, to determine its stability under the oxidative reforming of propane. Figure 7 shows that the H_2_ and CO production rates were stable for about the first 6 h. The H_2_ production and the CO production during the stability test were 2.37 mL·min^−1^·cm^−2^ and 0.77 mL·min^−1^·cm^−2^, respectively. Moreover, the H_2_/CO molar ratio was about 3.4 during the reaction time. After, a rapid decline in syngas production was observed. This fact is believed to be due to catalyst poisoning by carbon deposition. TGA analysis was performed to elucidate this fact, and the results show significant differences between the fresh and spent catalysts (Figure 8). Assuming that the observed weight loss in the TGA curve was due to the carbon combustion, the carbon content was, therefore, negligible for the fresh catalyst. In contrast, the spent catalyst showed a high total carbon content of 28 wt. %. These results corroborate that the observed hydrogen production involves oxidative reforming reactions and a significant contribution from the catalytic thermal cracking. Therefore, the latter reactions consumed the excess propane supply to the system, wherein the thermal decomposition of the possible intermediate products, as in Reactions (5)–(7), leads to high H_2_/CO molar ratios. In this sense, the propane concentration in the sweep and the stability of Ni-CeO_2_ catalysts need further investigation to enhance the obtainment of syngas through the proposed oxidative reforming of propane approach.

(5) $$C_{3}H_{8}\to 3C+{4H}_{2}∆H^{{}^{\circ}}=119.5\;{kJ\cdot mol}^{-1}$$

(6) $$C_{2}H_{4}\to 2C+{2H}_{2}∆H^{{}^{\circ}}=-52.5\;{kJ\cdot mol}^{-1}$$

(7) $$CH_{4}\to C+{2H}_{2}∆H^{{}^{\circ}}=74.85\;{kJ\cdot mol}^{-1}$$

**Figure 9:**
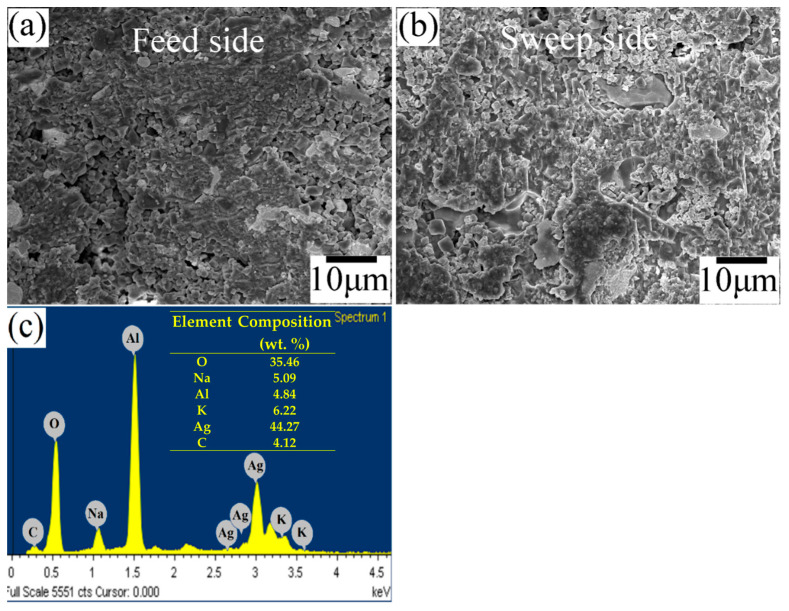
SEM postmortem of the cermet–molten-carbonate membrane: (**a**) feed-side surface in contact with the feed mixture of CO_2_/O_2_/N_2_ (15/17/68 vol. %); (**b**) surface in contact with a 5 vol. % of C_3_H_8_ in argon, at a total flow of 50 mL·min^−1^; (**c**) elemental analysis by EDS of the sweep side of the membrane.

After studying the oxidative reforming, the membrane surface was analyzed by SEM. Figure 9 shows the results of the postmortem analysis for both sides of the membrane. The surface that was in contact with C_3_H_8_ exhibited significant changes in its microstructure, primarily related to a growth in the size of the grains and porosity, while the surface that was in contact with the feed mixture of CO_2_/O_2_/N_2_ (15/17/68 vol. %) only showed minor microstructural changes. Although propane promotes certain changes on the membrane’s surface, its stability is remarkable because no N_2_ leaks were attributed to the membrane’s failure, nor were there significant fluctuations in the permeation performance. Finally, elemental analysis by EDS of the sweep side of the used membrane (Figure 9c) corroborates the presence of all of the original elements constituting the fresh membrane (Figure 3c). Overall, the results suggest the high potential of the proposed cermet–molten-carbonate membrane to couple the high-temperature CO_2_ and O_2_ separation with the oxy-CO_2_ reforming of propane as a potential conversion and valorization alternative for CO_2_.

## 4. Conclusions

A ceramic–carbonate membrane reactor was successfully probed to simultaneously perform the selective separation of CO_2_ and O_2_ at elevated temperatures with the subsequent production of syngas by coupling the oxidative reforming of propane. The results are conclusive and evidence the high capability of the studied ceramic–carbonate membrane to combine its perm-selectivity properties, exhibited between 725 and 850 °C, to perform the proposed catalyzed reactions. The studied operating temperatures promote the contribution of propane cracking to the desirable dry reforming and partial oxidation of the propane reactions. Indeed, as the catalyst’s deactivation resulted in the syngas production’s detriment, it is clear that further improvements must be made to the catalyst’s selection, looking to the future achievement of a higher carbon deposition resilience, especially over long-term operations.

## Data Availability

The original contributions presented in the study are included in the article/Supplementary Materials, further inquiries can be directed to the corresponding author.

## Supplementary Materials

The following supporting information can be downloaded at: https://www.mdpi.com/article/10.3390/membranes14110238/s1, S1: Conversion calculations.
