# Supplementary material for: Syngas Production via Oxidative Reforming of Propane Using a CO2- and O2-Permeating Membrane
Source: Membranes (Basel). 2024 Nov 12;14(11):238. doi: 10.3390/membranes14110238 (PMC11596156; doi:10.3390/membranes14110238)
Supplement: Supplementary file 1 [file membranes-14-00238-s001.zip › membranes-3264934-supplementary.pdf]

membranes

MDPI

Supplementary Materials

Syngas Production via Oxidative Reforming of Propane Using a CO2- and O2-Permeating Membrane

José A. Fabián-Anguiano [1], Lorena G. Cuéllar-Herrera [1], José A. Romero-Serrano [1], Issis C. Romero-Ibarra [2], Antonieta García-Murillo [3], Felipe Carrillo-Romo [3] and José Ortiz-Landeros [1,*]

1 Instituto Politécnico Nacional, Escuela Superior de Ingeniería Química e Industrias Extractivas, Departamento de Ingeniería en Metalurgia y Materiales, UPALM-Zacatenco, IPN Avenue, Mexico City 07738, Mexico
2 Instituto Politécnico Nacional, Unidad Profesional Interdisciplinaria en Ingeniería y Tecnologías Avanzadas, IPN Avenue, Mexico City 07340, Mexico
3 Instituto Politécnico Nacional, Centro de Investigación e Innovación Tecnológica, Cerrada de Cecati S/N, Santa Catarina, Azcapotzalco, Mexico City 02250, Mexico
* Correspondence: jortizla@ipn.mx; Tel.: +52-5557296000 (ext. 54267)

Based on the chromatography analysis, the conversion rate of C_3H_8, CO_2, and O_2 can be estimated by Eqs. (S1), (S2) and (S3).

$$X_{C_3H_8} = \frac{F_{C_3H_8(in)} - F_{C_3H_8(out)}}{F_{C_3H_8(in)}} \times (100) \quad \text{(S1)}$$

$$X_{CO_2} = \frac{J_{CO_2} - J_{CO_2\,(unconsumed)}}{J_{CO_2}} \times (100) \quad \text{(S2)}$$

$$X_{O_2} = \frac{J_{O_2} - J_{O_2\,(unconsumed)}}{J_{O_2}} \times (100) \quad \text{(S3)}$$

In Eq. (S1), $F_{C_3H_8\,(in)}$ and $F_{C_3H_8\,(out)}$ are the propane flow rate at the sweep in and the sweep out of the membrane reactor, while in Eq. (S2) and (S3) J_{CO_2}, J_{O_2} correspond to the permeated concentration of species through the membrane and J_{CO_2}, $J_{O_2\,(unconsumed)}$ represent the unreacted flux of CO_2 and O_2.
